# Expression of Matrix Metalloproteinase-21 in Oral Squamous Cell Carcinoma

**DOI:** 10.7759/cureus.34256

**Published:** 2023-01-27

**Authors:** Adhithya Baskaran, A Santhadevy, Nagaraj Vezhavendhan, Muthandam Sivaramakrishnan, Vidya Lakshmi Santhanam, Rajaram Suganya

**Affiliations:** 1 Oral & Maxillofacial Pathology and Oral Microbiology, Adhiparasakthi Dental College and Hospital, Chennai, IND; 2 Oral & Maxillofacial Pathology and Oral Microbiology, Indira Gandhi Institute of Dental Sciences, Pondicherry, IND

**Keywords:** mmp-21, oral cancer, immunohistochemical, oral squamous cell carcinoma, matrix metalloproteinase-21

## Abstract

Introduction

Oral squamous cell carcinoma (OSCC) is one of the most common cancer with increasing morbidity and mortality due to regional invasion, metastasis, and secondary malignancy along with associated medical complications. The degradation of matrix metalloproteinases (MMPs) is essential for the progression of carcinoma, hence MMPs are implicated in cancer invasion and metastasis as a biomarker.

Objective

The primary objective of this study was to find the expression of MMP-21 between oral squamous cell carcinoma and healthy tissue.

Materials and methods

In this retrospective study, banked pathology was used to observe the expression of MMP-21 in oral squamous cell carcinoma. MMP-21 protein expression was assessed in 50 cases of OSCC by immunohistochemistry assay. Statistical analysis was performed to evaluate the relation of MMP-21 expression.

Results

There was increased expression of MMP-21 in OSCC, and this was statistically significant (p<0.0001).

Conclusion

This result suggests the potential role in tumor progression and as a potent marker in diagnosing OSCC. In the future, it might also be a novel tool in therapeutic intervention.

## Introduction

Oral cancer is a neoplasia that arises in the oral cavity; nearly 90% of malignant carcinomas arise from the squamous epithelial cells, meaning they are referred to as squamous cell carcinomas [1,2]. Descriptions of cancers have been found in the ancient ruins of the Egyptian civilization of 3000-1600 BC [3]. Cancer is a generic term encompassing a large group of diseases that can affect any body part [4]. Head and neck cancers represent 5.3% of all cancers, around six lakh new cases of cancer in the oral cavity and pharynx are present, and it is estimated that more than three lakh deaths occur each year; these figures will nearly double by 2040 [5-7]. Early diagnosis increases the chances of survival and better prognosis [8]. There are several techniques for diagnosing oral squamous cell carcinomas (OSCC) which include (i) oral biopsy, (ii) saliva-based oral cancer diagnosis, (iii) imaging test, and (iv) biomarkers [9]. The first Immunohistochemical study was reported in 1941 by Coons et al., who used fluorescein isothiocyanate as an antibody and made it conjugate with an antigen of pneumococcus by the fluorescent dye in lesional tissues [10,11]. The biopsy tissue is used for diagnosis; with the support of molecular techniques such as assays and biomarkers, the prognosis of the diseases can be assessed, which helps predict the progression and treatment resistance of the disease. Likewise in immunohistochemistry, with the help of biomarkers, we can determine the diagnosis of the disease, drug development, and biological research [12,13].

Matrix metalloproteinases (MMPs) constitute a gene family whose corresponding enzymes are highly capable of degrading extracellular matrix (ECM) components that play a crucial role in tumor progression. MMP-21 studies show positive expression in development, stromal remodeling, and inflammation in tissue and high expression in gastric, esophageal, and colorectal squamous cell carcinomas [14-16]. To our knowledge, the association of MMP-21 expression in OSCC has not yet been addressed, so we estimated the protein expression of MMP-21 in OSCC specimens in the present study.

## Materials and methods

The work was approved by the Institutional Ethical Committee No. IGIDSIEC2018NRP21PGADOPM. The archival samples from Indira Gandhi Institute of Dental Science, Pondicherry, which was diagnosed as OSCC, were taken. The archival sample consists of 50 OSCC (Group A). Thirty normal gingival tissues, which were free of lesions, were used as the control group (Group B). MMP-21 was isolated from rabbits, and the antibody was diluted with a Phosphate buffer solution (1:50).

First, 4µ-thick sections were taken from the tissue embedded in paraffin samples, and these sections were deparaffinized and rehydrated. For retrieval of antigens, those tissue sections were immersed inside a Tris-buffer solution and warmed up at a temperature of 400°C in a Multiepitope retrieval system. Next, PolyExcel H_2_O_2_ was added to the section for endogenous peroxidase blocking for 15 minutes. Thereafter, indefinite binding sites were obstructed with normal serum. Then incubation was processed in tissue samples with MMP-21 polyclonal antibodies in a moist chamber for one hour. With a wash buffer, the sections were washed in buffer solution for five minutes, with two changes. Then a secondary antibody PolyExcel HRP (DAKO) solution was put into the tissue sections and incubated for 20-30 minutes. Then diaminobenzidine's (DAB's) working solution (DAB buffer - 1 ml + DAB chromogen - one drop) was added to the section in a dark chamber, acting as the primary stain. Hematoxylin was used as the counterstain for 10-30 seconds. Dehydration of sections was done with an increased gradation of alcohol and then cleared by immersing in xylene. Sections were parched and mounted using dibutylphthalate polystyrene xylene (DPX). A tissue section was made from paraffin-embedded breast cancer tissue and used as a positive control; these samples were done in the same manner as the test groups.

The details of the sectioned tissue specimens were blindfolded and evaluated by two pathologists. The evaluation was done based on the immunoreactivity score (IRS) system, viewed under high magnification. Based on the IRS criteria, it was classified as (A) & (B), (A) if the number of positively stained cells was < or = 5%, it scored 0; if stained positive cells were around 6-25%, it scored one; if stained positive cells were 26-50%, it scored two; if stained positive cells were 51-75%, it scored three; if stained positive cells were 75%, it scored four. (B) Based on the intensity of the stain, if it was colorless it scored 0; on a mild reaction on the stain, it scored one; on a moderate reaction, it scored two; on an intense reaction, it scored three. By multiplying A and B, the overall staining score was stratified as 0 to one for negative, two to four for weak, four to eight for moderate, and nine to twelve for strong according to the proportion and intensity of positively stained cancer cells. If the difference in scores from two pathologists was more than three, rescoring was done. The specimens scored as mild, moderate, and strong positive staining, it was combined as a positive group.

Statistical analysis

Correlations between immunohistochemical staining of MMP-21 and clinicopathological characteristics with differences of P value 0.05 or less were considered to be statistically significant. 

## Results

**Figure 1:**
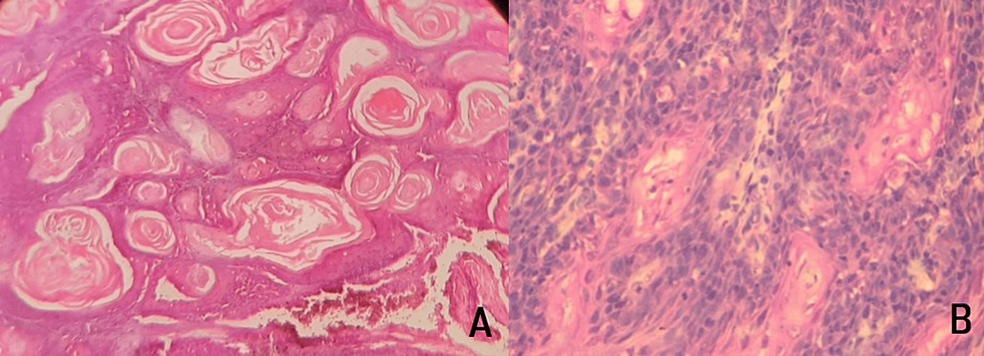
Histopathological image of Oral Squamous cell carcinoma H&E stain (A) 4x & (B) 40x

**Figure 2:**
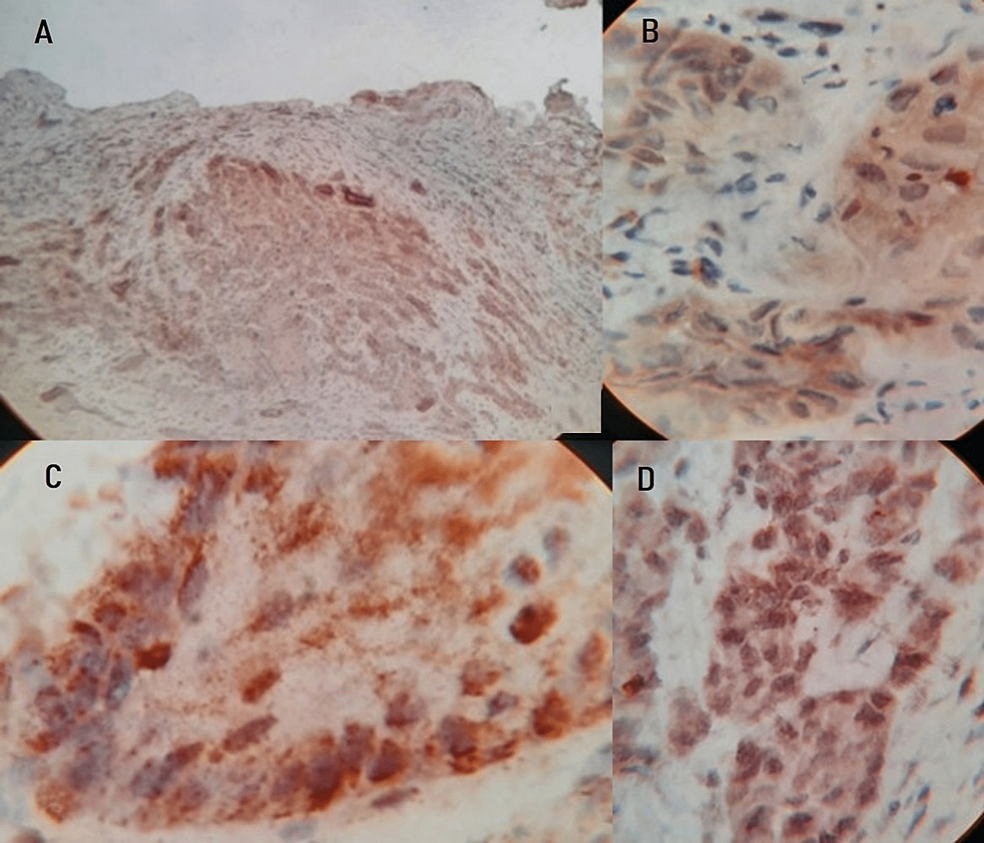
Histopathological image shows Oral Squamous cell carcinoma - IHC stain (A) 4x, (B) 40x & (C), (D) 100x

In this study, an investigation of 50 cases of OSCC tissues (Figure 1) was incorporated. MMP-21 expression is found in the cytoplasm and cell membrane of tumor cells. The standard IRS system was applied to the staining of MMP-21, among those 46 cases were identified as positive staining (Figure 2) and four cases were identified as negative staining (-). In contrast, among matched normal tissues, 30 cases were designated as negative staining (Table 1). The statistical analysis revealed that the association between immunohistochemical staining of MMP-21 in normal and OSCC samples was found to be statistically significant with p<0.0001, this indicates that MMP-21 expression in OSCC was significantly increased compared with that in normal tissues with a mean percentage of 64.28 and Standard Deviation of 24.13.

**Table 1:** Grade percentage of MMP-21 between OSCC and normal gingiva.

MMP-21		
GRADE	OSCC (50 cases)	Normal Gingiva (30 Cases)
Positive Expression	92%	Nil
Negative Expression	8%	100%
Total	100%	100%

## Discussion

In India, OSCC accounts for most oral malignant tumors, bearing more than one-third of the global burden [16, 17]. When accounting for OSCC’s poor prognosis, it is widely accepted that it has the capability to invade adjacent regions and is prone to metastasize into lymph nodes and distant organs.

Connective tissue is predominantly composed of the ECM, which is a three-dimensional network consisting of extracellular macro molecules and minerals such as collagen, enzyme, glycoprotein, and hydroxyapatite that provide biochemical and structural support to the surrounding tissues. ECM is an invasive component of the tumor microenvironment and cancer development progression and can be related to increased extracellular matrix deposition. The ECM not only influences malignancy and growth of tumors but also has effects on therapy. ECM must be explored completely to develop a positive outcome and to reduce negative effects.

The ECM has an advantage in the invasion of tumors; tumor cells have the potential to produce proteolytic enzymes that directly affect their metastatic behavior [18]. Recently, molecular-based biomarkers have been used for early diagnosis and better prognosis. MMP-21 is one such biomarker that encodes members of MMPs’ family protein. MMPs are a degradation protein of ECM that constitute a family of proteolytic enzymes that are structurally connected but genetically different, and they act on the turnover and degradation of extracellular matrix proteins, MMP-21 in the family, as used in the breakdown of ECM for both physiological processes such as tissue remodeling and embryonic development and disease processes such as metastasis. The currently used clinical staging system tumor, node, metastasis (TNM) does not provide adequate differentiation and characterization of patients with malignant tumors. Assessment of early diagnosis (Stage I-II) corresponds to a vastly improved five-year survival rate over a more advanced stage (Stage III-IV). Although a variety of molecular markers have been used to diagnose squamous cell carcinoma, sensitive and specific biomarkers play a vital role in improving the lifestyle of a patient. For this purpose, we investigated the expression of MMP-21 in OSCC.

The expression of MMP-21 was analyzed in OSCC cases and statistical correlation was assessed with their expressions in OSCC. Based on an immunohistochemistry assay and staining score system, we found that MMP-21 protein expression was significantly increased in OSCC compared with normal tissue, indicating its role in MMP-21 in diagnosing OSCC. Out of 50 cases, 46 cases expressed MMP-21, and four cases did not show or poorly expressed MMP-21. The 30 gingival tissues that were used as a control group did not show any expression for MMP-21. Obtained P-value score <0.0001 was statistically significant.

On tissue injury, the expression of MMP-21 was decreased in keratinocytes, which was because only transforming growth factor b1 can induce MMP-21 expression in keratinocytes. MMPs are primarily expressed in stromal cells in vivo; the expression of MMP-21 is largely restricted to epithelium and tumor cells. The expression of MMP-21 is noticed in the invasive front of the tumor rather than the dysplastic cells. In addition, the expression of MMP-21 is also found to be expressed in macrophages and fibroblasts both in vivo and in vitro [19]. Wu T et al. stated that MMP-21 expression in gastric cancer was associated with tumor invasion and metastasis [20]. Zhao et al. also demonstrated a strong association between MMP-21 expression and esophageal squamous cell carcinoma (ESCC) invasion status, metastasis, and TNM stage [15]. Previous researchers have found that MMP-21 has the capability to degrade denatured collagen, especially collagen type IV, V, VII, IX, and X; thus, MMP-21 eases tumor cells’ invasion and facilitates metastasis. In patients with ESCC, the MMP-21 expression could serve as an independent prognostic factor; this might be ascribed to its ECM-degrading capacity, which has a potential role in the mechanism of oncogenic activity. It was found that positive staining of MMP-21 was detected in ESCC and positively correlated with deep tumor invasion, metastasis to lymph node, distant metastasis, and advanced TNM stage, which suggests that MMP-21 plays a promotive role in invasion and metastasis. Expression of MMP-21 in gastric, esophageal, and colorectal squamous cell carcinoma was estimated to be 60-70%, and the present study of OSCC shows strong expression of MMP-21 [18,21].

To our best knowledge to compare our values, there was there are no other articles whose authors estimated the expression of MMP-21 in OSCC by Immunohistochemical method. There may be some possible limitations in this study as we have used polyclonal antibodies, further studies are required using monoclonal antibodies with increasing the higher sample size and including different grades of OSCC can provide the establishment of an association between MMP-21 and OSCC.

## Conclusions

Our data revealed a significantly increased expression of MMP-21 in OSCC, this suggests that MMP-21 can be used to identify OSCC cells. Based on the current findings we conclude that MMP-21 is very likely to afford an indication of invasion of tumor and it could be an independent prognostic marker for patients with OSCC. We believe that MMP-21 can be a diagnostic marker and act as a potential therapeutic target for patients with OSCC.

## References

[1] Rivera C (2015). Essentials of oral cancer. Int J Clin Exp Pathol.

[2] International Agency for Research on Cancer (2017). WHO classification of head and neck tumours.

[3] Inchingolo F, Santacroce L, Ballini A (2020). Oral cancer: a historical review. Int J Environ Res Public Health.

[4] Kademani D (2007). Oral cancer. Mayo Clin Proc.

[5] https://www.who.int/cancer/prevention/diagnosis-screening/oral-cancer/en.

[6] (2022). WHO report on cancer: setting priorities, investing wisely and providing care for all. https://www.who.int/publications/i/item/9789240001299.

[7] Aupérin A (2020). Epidemiology of head and neck cancers: an update. Curr Opin Oncol.

[8] https://www.cancerresearchuk.org/about-cancer/cancer-symptoms/why-is-early-diagnosis-important.

[9] Omar EA (2013). The outline of prognosis and new advances in diagnosis of oral squamous cell carcinoma (OSCC): review of the literature. Journal of Oral Oncology.

[10] Duraiyan J, Govindarajan R, Kaliyappan K, Palanisamy M (2012). Applications of immunohistochemistry. J Pharm Bioallied Sci.

[11] Coons AH, Creech HJ, Jones RN (2016). Immunological properties of an antibody containing a fluorescent group. Proceedings of the society for experimental biology and medicine.

[12] Ziv E, Durack JC, Solomon SB (2016). The importance of biopsy in the era of molecular medicine. Cancer J.

[13] https://www.who.int/news-room/fact-sheets/detail/cancer.

[14] Tsukifuji R, Tagawa K, Hatamochi A, Shinkai H (1999). Expression of matrix metalloproteinase-1, -2 and -3 in squamous cell carcinoma and actinic keratosis. Br J Cancer.

[15] Zhao Z, Yan L, Li S, Sun H, Zhou Y, Li X (2014). Increased MMP-21 expression in esophageal squamous cell carcinoma is associated with progression and prognosis. Med Oncol.

[16] Kusukawa J, Sasaguri Y, Morimatsu M, Kameyama T (1995). Expression of matrix metalloproteinase-3 in stage I and II squamous cell carcinoma of the oral cavity. Journal of oral and maxillofacial surgery.

[17] Tandon A, Bordoloi B, Jaiswal R, Srivastava A, Singh RB, Shafique U (2018). Demographic and clinicopathological profile of oral squamous cell carcinoma patients of North India: A retrospective institutional study. SRM Journal of Research in Dental Sciences.

[18] Mishev G, Deliverska E, Hlushchuk R, Velinov N, Aebersold D, Weinstein F, Djonov V (2014). Prognostic value of matrix metalloproteinases in oral squamous cell carcinoma. Biotechnol Biotechnol Equip.

[19] Skoog T, Ahokas K, Orsmark C, Jeskanen L, Isaka K, Saarialho-Kere U (2006). MMP-21 is expressed by macrophages and fibroblasts in vivo and in culture. Exp Dermatol.

[20] Wu T, Li Y, Lu J (2013). Increased MMP-21 expression is associated with poor overall survival of patients with gastric cancer. Med Oncol.

[21] Pu Y, Wang L, Wu H, Feng Z, Wang Y, Guo C (2014). High MMP-21 expression in metastatic lymph nodes predicts unfavorable overall survival for oral squamous cell carcinoma patients with lymphatic metastasis. Oncol Rep.

